# Antiproliferative activity of *Moringa oleifera* (L.) Lam. and *Moringa stenopetala* (Bak.) Cufod. leaves extract against selected cancer cells in primary cell culture

**DOI:** 10.1038/s41598-025-33559-6

**Published:** 2025-12-23

**Authors:** Masresha Ahmed Assaye, Poupak Fallahi, Duccio Volterrani, Silvia Martina Ferrari, Emilio Barozzi, Armando Patrizio, Hagos Tesfay, Solomon Genet Gebre

**Affiliations:** 1 Department of Internal Medicine, School of Medicine, College of Health Sciences, Addis Ababa University, Addis Ababa, Ethiopia; 2 Department of Medical Biochemistry, School of Medicine, College of Health Sciences, Addis Ababa University, Addis Ababa, Ethiopia; 3 Department of Translational Research and of New Technologies in Medicine and Surgery, University of Pisa, Pisa, Italy; 4 Department of Clinical and Experimental Medicine, University of Pisa, Pisa, Italy; 5 Department of Surgical, Medical and Molecular Pathology and Critical Area, University of Pisa, Pisa, Italy; 6 Department of Emergency Medicine, Azienda Ospedaliero-Universitaria Pisana, Pisa, Italy

**Keywords:** Antiproliferative, *Moringa oleifera*, *Moringa stenopetala*, Cancer, Primary cell culture, Cancer, Drug discovery, Oncology

## Abstract

Natural compounds possess anti-tumor capabilities for a wide range of cancers. The present study aims to analyze and compare the antiproliferative activity of *Moringa oleifera* and *Moringa stenopetala* leaf extracts against anaplastic thyroid carcinoma and lung adenocarcinoma cells in primary cell culture. Samples were collected from Arba Minch, Ethiopia. Water-soluble tetrazolium salt-1 assay was used to assess the antiproliferative activity of these samples against anaplastic thyroid carcinoma and lung adenocarcinoma cells in primary cell culture. Cell number counting was another method used to confirm cell proliferation. The results showed that, in comparison to the control, both *Moringa* species significantly lowered cancer cell proliferation in a dose-responsive manner. Although *Moringa oleifera* showed a stronger inhibitory effect compared to *Moringa stenopetala*, there was no statistically significant difference (*p* > 0.05) in the proliferation of anaplastic thyroid carcinoma and lung adenocarcinoma cells between the two species. *Moringa oleifera* extract had a lower IC_50_ value in anaplastic thyroid carcinoma cells, 84.2 µg/ml, than *Moringa stenopetala* leaf extract, 90.6 µg/ml. *Moringa oleifera* leaf extract has a lower IC_50_ value in lung adenocarcinoma cells, 64.7 µg/ml, than *Moringa stenopetala* leaf extract, 75.3 µg/ml. In conclusion, the two species have antiproliferative activity on anaplastic thyroid carcinoma and lung adenocarcinoma cells in primary cell culture. These findings indicated that both extracts may serve as a basis for future cancer therapeutic applications.

## Introduction

Cancer is a disease in which cells from any part of the body behave inappropriately and multiply uncontrollably. Cancer progression is complex and multi-regulatory, making it a difficult disease to treat and survive^1^. It is projected to be one of the leading causes of death globally in 2020, with 10 million deaths and 19 million new cases^2^. By 2040, 28.4 million cases of cancer will occur globally, according to GLOBOCAN forecasts^3^.

Anaplastic thyroid cancer (ATC) represents only 2%–3% of cases of thyroid cancer, and it is characterized by extreme aggressiveness, with a death rate close to 100%. and there is currently no pharmacological medication capable of treating this illness^4^. Lung cancer is the leading cause of cancer incidence and mortality globally^5^. Lung adenocarcinoma (LA) accounts for approximately 40% of all lung malignancies and arises from the mucosal glands^6^. At different stages of the disease, several medicines have been utilized to treat lung cancer. But these drugs frequently cause patients to experience varied degrees of side effects^7^.

Modern cancer treatments like radiation and chemotherapy can kill cancer cells but also result in side effects and resistance. Thus, alternative therapeutic options are required to address side effects and resistance. Natural compounds derived from plants offer a promising solution, as they have demonstrated the ability to successfully overcome cancer drug resistance but also serve to enhance anti-tumor effects and reduce systemic toxicity^1^. Many traditional medicinal plants, however, have not yet undergone a thorough scientific evaluation as possible anticancer therapeutic agents^8^.

Within the *Moringaceae* family, there is only one genus: *Moringa.* The genus *Moringa* contains fourteen species; two commonly cultivated members of the *Moringaceae* family are *M. stenopetala* (Baker f.) Cufod and *M. oleifera* Lam.^9,10^. *M. stenopetala* is also known as the African *Moringa* tree due to its widespread distribution in southern Ethiopia, eastern Somalia, and northern Kenya. In Ethiopia, it has several names: Halako in Gofa and Wolayita, Shiferaw in Amharic, Shelagta in Konso, and Haleko in Derashe^11,12^. In southern Ethiopia, the local communities prepare the leaves as cabbage and consume them with “*Kurkurfa*,” a traditional dish^11,13^. *M. oleifera* is one of 14 species of the genus *Moringa*. It is the most well-known and studied. It is found in several subtropical and tropical countries^14,15^.

*Moringa* leaves provide several health advantages, including anti-inflammatory, anti-cancer, anti-diabetic, and antibacterial properties^16^. Numerous scientific studies have shown that *M. stenopetala* and *M. oleifera* contain many bioactive compounds, including alkaloids, flavonoids, glycosides, and phenolic compounds, determining the antioxidant, anti-inflammatory, and anti-cancer^10,17^. Phenolic compounds that demonstrated a substantial decrease in cancer cell proliferation were identified by Reverse Phase–High Performance Liquid Chromatography (RP-HPLC) profiling of *M. oleifera* leaf extract. These compounds included quercetin, caffeic, m-coumaric, ferulic, 4-hydroxy 3-methoxy cinnamic, gallic, p-coumaric, sinapic, syringic, 4-hydroxybenzoic, and vanillic acids in the chromatogram^18^. In an Egyptian investigation, Liquid Chromatography–Quadrupole Time-of-Flight Mass Spectrometry (LC-qTOF-MS) profiling was performed on the methanol extract of *M. stenopetala;* 50 compounds, including terpenes (such as loliolide and dihydroactinidiolide), niazirin, quinic acid derivatives, and flavonoids, had been identified with significant cytotoxic effects^19^.

Geographical location and cultivar affect the nutritional value of *Moringa*^14^. Plants of the same species grown under various conditions may differ significantly in the quantity of bioactive chemicals they contain^20,21^. Changes in a plant’s origin or variety can lead to different metabolite phenotypes because these factors affect the makeup of the plant’s metabolites. The amount and composition of the bioactive compounds in a plant affect its pharmacological characteristics^22^. The anti-cancer properties of these two Ethiopian-grown *Moringa* species have not been studied. Furthermore, little international research has been done on *M. stenopetala’s* anti-cancer effects. As far as we are aware, no previous research has looked at the effect of two *Moringa* species on ATC cells. Additionally, the effect of the two *Moringa* species on primary cell cultures has not been previously reported. As a result, the purpose of this study is to fill all of these gaps by evaluating the antiproliferative activity of these two species in primary cell culture. Furthermore, the study compares the antiproliferative activity of *M. stenopetala* to the well-studied *M. oleifera*.

## Methods and materials

### Source of *Moringa* leaves

Fresh leaves of the two species were collected from Arba Minch, Ethiopia. It is located at 6°01’59” N and 37°32’59” E, at an altitude of 1269 m above sea level, receives an average annual rainfall of 952.1 mm, and is 505 km away from Ethiopia’s capital, Addis Ababa^23^. The plants were authenticated by a taxonomist (Mr. Melaku Wondafrash), and a voucher specimen of each plant material was deposited at the National Herbarium, College of Natural Sciences, Addis Ababa University, for future reference with voucher numbers MA001 and MA002 for *M. oleifera* and *M. stenopetala*, respectively.

### Sample collection and preparation

A collection of fresh, mature, and healthy *Moringa* leaves from each species was made and brought to the laboratory for further processing. The leaves were sequentially washed with tap water, distilled water, 70% ethanol (1 min), and 1% sodium hypochlorite (5 min). After that, sterile distilled water was used to thoroughly rinse them^24,25^. To get rid of the extra moisture, the cleaned leaves were spread out on a spotless surface and dried in a shaded area. These steps play a substantial role in the removal of dust, pathogens, and microbes from the leaf’s surface, which is greatly aided by this step^26^. The leaves were ground in an electric multifunction mill (HYDDNice: Model HY-AB-0326, China) and then passed through a screen (20 mesh). Finally, it was stored in a refrigerator at -20 °C before being utilized.

### Preparation of *Moringa* leaves extract

Five grams of each powder and one hundred milliliters of methanol were combined to form methanolic dry extracts of the two species, and then two sonication cycles (40 °C, 60 min, 80%) and centrifugation for nine minutes at 3500 rpm were applied. (using a Beckman Coulter Allegra 21R refrigerated centrifuge, USA). Whatman No. 1 filter paper was utilized to filter the extract. In the vacuo rotary evaporator (Buchi, R-210, Switzerland), the filters from the two cycles were combined and concentrated. Before being used, the extracts were sealed, labeled, and stored at -20 °C in dark containers^27,28^. Since methanol is more polar than other forms of alcohol like ethanol, acetone, etc., and because methanol extraction frequently yields the greatest extraction results, it was typically chosen as the organic solvent in this investigation. In particular, using sonication for extracting leaves under various conditions^27^. The extraction yield was calculated as shown in the equation below^29^.

$${\text{Extraction yield }}\left( \% \right){\text{ }}=\frac{{{\text{Mass of extract}}}}{{{\text{Mass of the sample powder}}}} \times 100$$

### Primary cell culture source

Primary cancer cell cultures (ATC and LA cells) were obtained from surgical samples of patients treated at Pisa University. All patients signed a voluntary agreement, which was approved by Pisa University’s Ethics Committee.

#### Primary ATC cell culture

In M-199 medium containing 500 IU/ml streptomycin, 1,000 IU/ml nystatin, and 500 IU/ml penicillin, surgical sample tissues were rinsed to cultivate primary cancer cells, then suspended in DMEM ( Dulbecco’s Modified Eagle’s Medium) with % w/v glutamine, 50 µg/ml penicillin/streptomycin, and 20% v/v FCS (Fetal Calf Serum) and maintained in 5% CO_2_ at 37 °C (reagents were bought from Merck, Sigma-Aldrich, Darmstadt, Germany)^30–32^. The cells were trypsinized and multiplied in tissue culture flasks once they had reached confluence. The biggest cells were amplified in flasks and seeded in Methocel at the third passage to assess the efficiency of colony formation^30^.

According to earlier reports, cells’ chemosensitivity was assessed at the fourth passage^33,34^. Primary ATC cells were cultured under standard conditions and expanded to the 4th passage (5 weeks) for chemosensitivity assays. Morphology, confluency, and pH stability were evaluated to track growth. After confluent cells were separated using trypsin and placed in tissue culture flasks, the effectiveness of colony-forming was assessed using methocel coating at passage 3. To get enough viable cells for repeatable analyses, the largest colonies were separated and grown.

#### Primary LA cell cultures

To cultivate primary LA cells, surgical sample tissues were thoroughly chopped and placed in DMEM with 1% w/v glutamine, 20% v/v FCS, and 50 µg/ml penicillin/streptomycin^35^.

### Water-soluble tetrazolium salt-1 assay (WST-1) for cell proliferation and viability

Cell proliferation and viability were assessed using the WST-1 assay (Roche Diagnostics, Almere, Netherlands). Methanolic crude extracts of *M. stenopetala* and *M. oleifera* were reconstituted in dimethyl sulfoxide (DMSO), with the final DMSO concentration in all treated and control wells maintained below 0.1% to prevent cytotoxic effects. ATC and LA cells were seeded in 96-well microtiter plates at a density of 35,000 cells/mL, with a final volume of 200 µL per well. For treatment, 20 µL of extract solution was added to each well, resulting in final concentrations of 50, 100, 250, and 500 µg/m. The doses were selected based on preliminary dose-response experiments conducted in our laboratory. Negative controls containing culture medium only, as well as DMSO controls, were included. Following incubation for 48 h at 37 °C in a humidified atmosphere with 5% CO₂, 10 µL of WST-1 reagent was added to each well and incubated for an additional 2 h at 37 °C. Using an ELISA reader, a multi-well spectrophotometer plate reader, OD was measured at 450 nm. The IC_50_ values were estimated by nonlinear curve-fitting. The percentage of cell viability that the extracts inhibited cell growth was used to calculate the extracts’ inhibitory effects, with untreated cells being 100% viable^30^.

In comparison, cell number counting was used to assess proliferation^28,31,36^.

### Cell counting

Cell number counting was another method used to confirm cell proliferation. WST-1 measures mitochondrial cell activity, and investigations have demonstrated that there is no clear correlation with cell number. With twenty-four well tissue culture plates, cells were seeded at a density of 13,000 cells per well in a medium containing 10% Fetal Calf Serum (FCS), with or without the necessary components. Every other day, the medium was changed. Following a 72-hour incubation period at 37 °C with 5% CO_2_, the cells were extracted from the plates using 500 mL of Phosphate-Buffered Saline (PBS) supplemented with 100 mg of trypsin and 1 mM EDTA. Extending the incubation allows sufficient time for differences in growth kinetics between treated and control cells to become measurable. The hemocytometer was used to count the cells^30^.

### Ethics statement

The study was approved by the Institutional Review Board (IRB) of the Addis Ababa University, College of Health Science (Meeting No. 10/2024, Protocol No. 050/23/Biochemistry) and the research ethics and review committee of the Department of Biochemistry, College of Health Science, Addis Ababa University (Ref. No. SOM/BCHM/108/2015). Primary cancer cell cultures (ATC and LA cells) were obtained from surgical samples of patients treated at Pisa University. All participants provided written informed consent before participating in the study. The study protocol and consent procedure were approved by the Ethics Committee of Pisa University. The studies were carried out in compliance with all local legislation and institutional requirements for medical research as well as ethical guidelines.

### Plant collection approval

Permission for the collection of *M. oleifera* and *M. stenopetala* leaves was granted by Arba Minch University, College of Agricultural Sciences, Arba Minch, Ethiopia (Approval Letter No. A/C/C1/5/157). Additionally, consent was obtained from the respective landowners for the collection and use of the plant materials for research purposes. All collection activities were conducted in accordance with relevant institutional, national, and international guidelines and regulations.

### IUCN policy statement

The collection of plant material complies with relevant institutional, national, and international guidelines and legislation.

### Statistical analysis

Every experiment was run through three tests, and the mean ± SD of the data was presented. Statistical analysis was performed using GraphPad Prism version 10.6.0 (GraphPad Software, San Diego, CA, USA; https://www.graphpad.com). The IC_50_ values were estimated by nonlinear curve-fitting. The results were examined using one-way ANOVA and Tukey’s multiple comparison test to assess group differences. To compare the differences between the two groups, a t-test was used. Any differences with *P <* 0.05 (^∗^), *P <* 0.01 (^∗∗^), or *P* < 0.001 (^***^) were considered statistically significant.

## Results

The percentage of extraction yield of all extracts was also calculated. The percentage extraction yield of *M. oleifera* and *M. stenopetala* was 24.5% and 24.7%, respectively.

### Antiproliferative activity of *M. oleifera* leaf extract on ATC cells

**Fig. 1:**
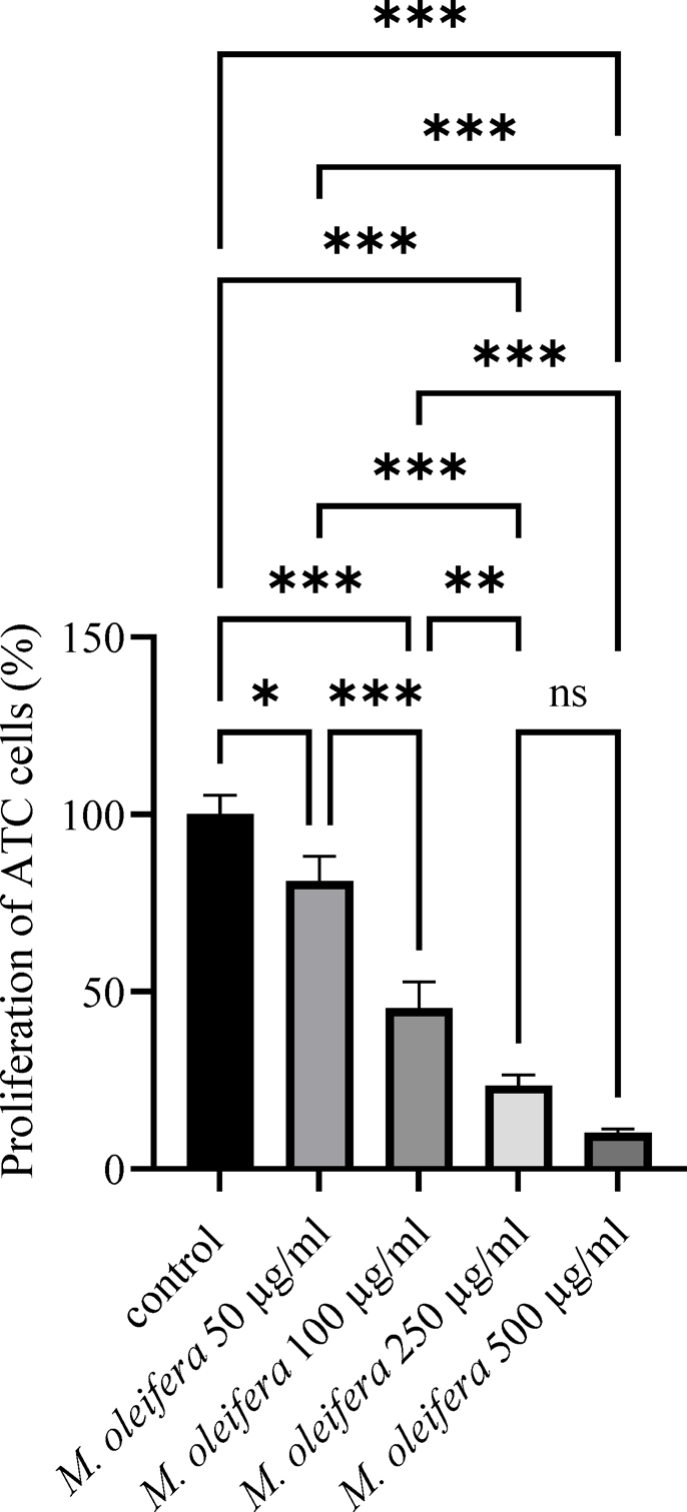
*M. oleifera* leaf extract activity on the proliferation of ATC cells. Values are presented as mean ± SD (*n* = 3). Statistical significance was determined using one-way ANOVA followed by Tukey’s post-hoc test. Differences were considered significant at *P < 0.05, **P < 0.01, and ***P < 0.001; ns indicates non-significant (*P* > 0.05).

It was demonstrated that, in comparison to the control, proliferation of anaplastic thyroid cancer cells decreased in a dose-responsive way with increasing extract concentrations (Fig. 1). Following exposure to the extract at concentrations of 50 µg/ml, 100 µg/ml, 250 µg/ml, and 500 µg/ml, the ATC cells’ proliferation dropped significantly to 81.2%, 50.9%, 23.5%, and 10.2%, respectively, in comparison to the control. *M. oleifera* leaf extract significantly reduces proliferation at all concentrations (*P* < 0.05 for 50 µg/ml and *P* < 0.001 for 100, 250, and 500 µg/ml). The cell count verified the above-mentioned findings. In the control group of anaplastic thyroid carcinoma cells, the number of cells was 18,800 ± 920/100 µl/well; among those administered *M. oleifera* extract with 50 µg/ml, 100 µg/ml, 250 µg/ml, and 500 µg/ml, the number of cells was 15,228 ± 790 (81%), 9,400 ± 960 (50%), 4,512 ± 780 (24%), and 1,880 ± 940 (10%), respectively (*P* < 0.01, ANOVA).

### Antiproliferative activity *M. stenopetala* leaf extract on ATC cells

**Fig. 2:**
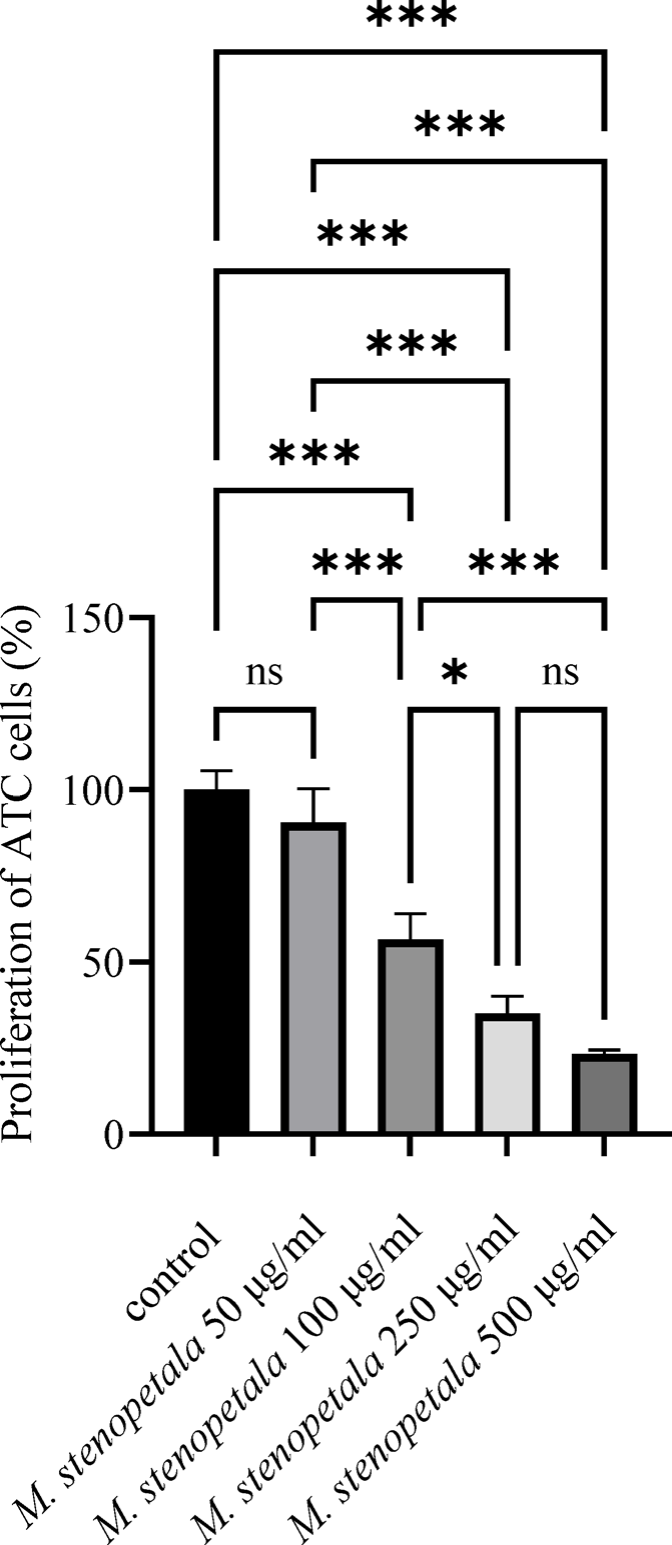
*M. Stenopetala* leaf extract activity on the proliferation of ATC cells. Values are presented as mean ± SD (*n* = 3). Statistical significance was determined using one-way ANOVA followed by Tukey’s post-hoc test. Differences were considered significant at *P < 0.05, **P < 0.01, and ***P < 0.001; ns indicates non-significant (*P* > 0.05).

As illustrated in Fig. 2, treatment with *M. stenopetala* leaf extract decreased the proliferation of ATC cells in a dose-dependent manner with increasing extract concentrations compared to the control. The results of the cell viability assay displayed that after *M. stenopetala* leaf extract treatment (50–500 µg/ml), the proliferation of anaplastic thyroid carcinoma cells significantly decreased to 90.5%, 56.6%, 35.1%, and 23.3%, respectively, compared to the control. The decrease in the percentage of proliferation becomes significant at higher doses, at a range of 100–500 µg/ml concentrations of *M. stenopetala* leaf extract (*P* < 0.001 for 100, 250, and 500 *μ*g/ml, respectively). The cell count verified the above-mentioned findings. In the control group of ATC cells, the number of cells was 18,800 ± 920/100 µl/well; in the group treated with *M. oleifera* leaf extract with 50 µg/ml, 100 µg/ml, 250 µg/ml, and 500 µg/ml, the number of cells was 17,108 ± 980 (91%), 10,716 ± 960 (57%), 6,580 ± 890 (35%), and 4,324 ± 930 (23%), respectively (*P* < 0.01, ANOVA).

### Antiproliferative activity of the two species on ATC cells

**Fig. 3:**
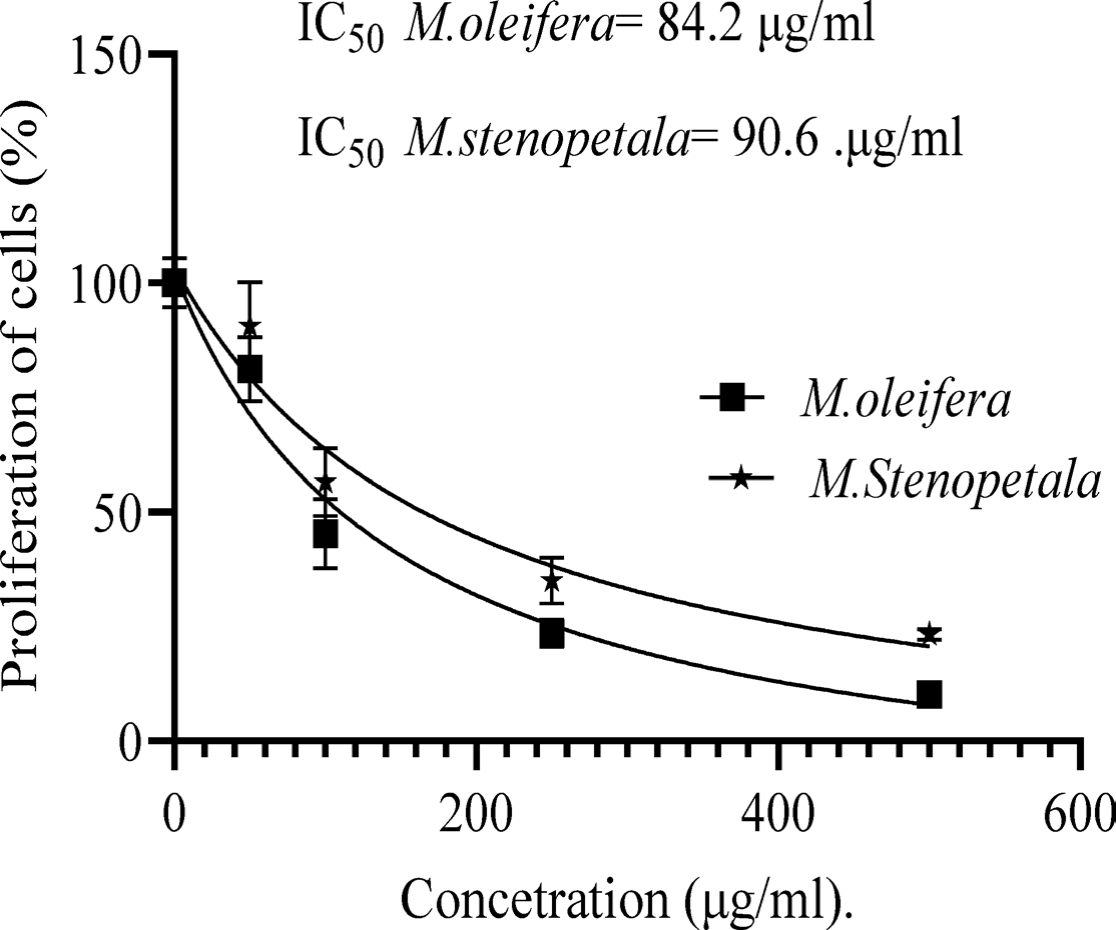
Comparative proliferation inhibition of *M. oleifera* and *M. stenopetala* on ATC Cells. Values are presented as mean ± SD (*n* = 3). IC_50_ values were determined after 48 h of exposure. Although Plant *M. oleifera* has a lower IC_50_ (84.2 µg/mL) than *M. stenopetala* (90.6 µg/mL), t-test revealed no significant difference (*P* > 0.05).

As illustrated in Fig. 3, increasing the concentration of both *Moringa* species resulted in a decrease in cancer cell proliferation. *M. oleifera* has a stronger effect than *M. stenopetala*, but statistical t-test analysis revealed no significant differences (*p* > 0.05) in the proliferation of anaplastic thyroid cancer cells between the two species. *M. oleifera* leaf extract had a lower IC_50_ value (84.2 µg/ml) in ATC cells compared to *M. stenopetala* leaf extract (90.6 µg/ml) after 48 h of exposure (Fig. 3). Because the cytotoxic effect of a sample increases with decreasing IC_50_ value, *M. oleifera* has a stronger cytotoxic effect than *M. stenopetala.*

### Antiproliferative activity *M. oleifera* leaf extract on LA cells

**Fig. 4:**
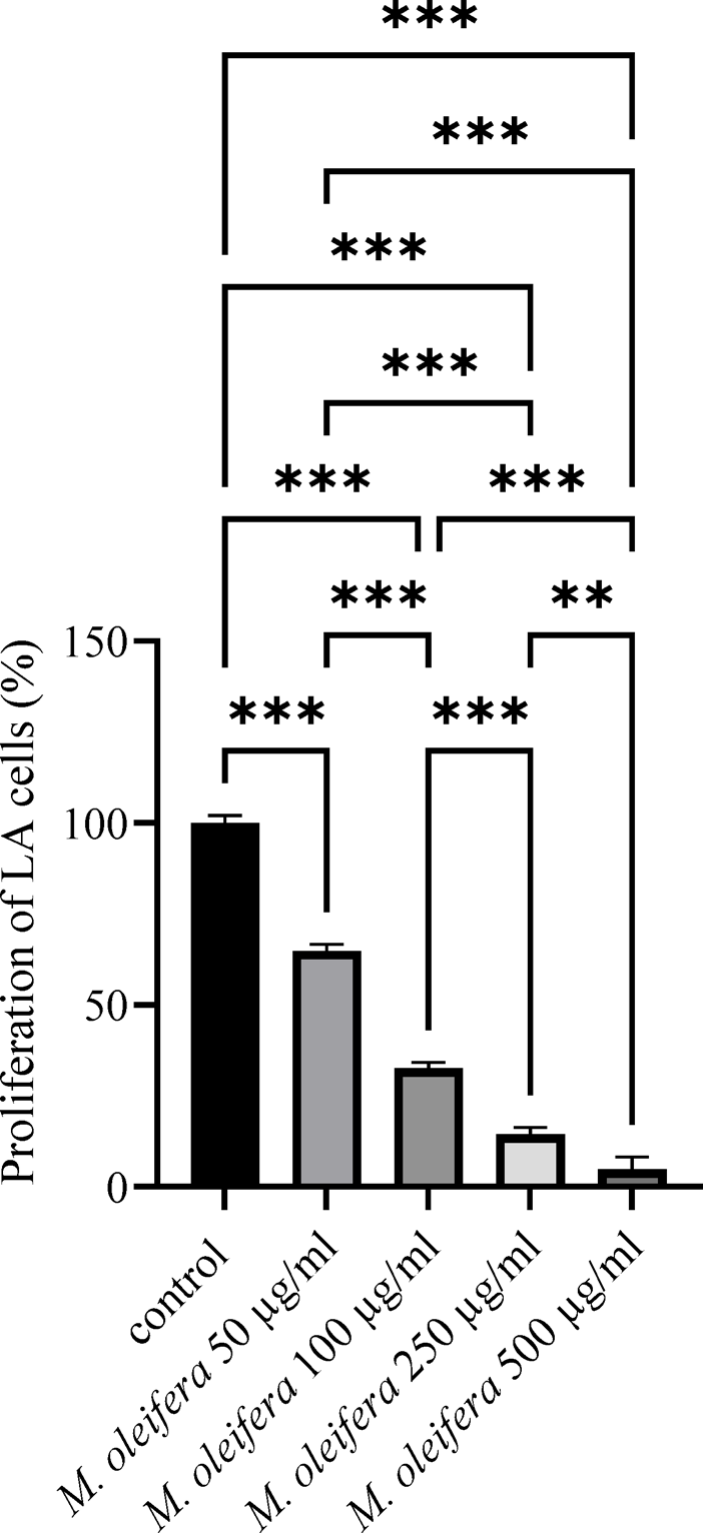
LA cell proliferation following exposure to different doses of *M. oleifera* leaf extract. Values are presented as mean ± SD (*n* = 3). Statistical significance was determined using one-way ANOVA followed by Tukey’s post-hoc test. Differences were considered significant at *P < 0.05, **P < 0.01, and ***P < 0.001.

It was demonstrated that, in comparison to the control, the proliferation of LA cells reduced in proportion to the increasing concentrations of the extract (Fig. 4). Following exposure to the extract at concentrations of 50 µg/ml, 100 µg/ml, 250 µg/ml, and 500 µg/ml, the LA cells’ proliferation dropped significantly to 64.4%, 32.5%, 14.5%, and 4.8%, respectively, versus the control group (*P* < 0.00). The cell count verified the above-mentioned findings. In the control group of LA cells, the number of cells was 18,800 ± 920/100 µl/well; in the group treated with *M. oleifera* with 50 µg/ml, 100 µg/ml, 250 µg/ml, and 500 µg/ml, the number of cells was 12,805 ± 980 (65%), 6,501 ± 860 (33%), 2,758 ± 650 (14%), and 985 ± 550 (5%), respectively (*P* < 0.01, ANOVA).

### Antiproliferative activity *M. stenopetala* leaf extract on LA cells

**Fig. 5:**
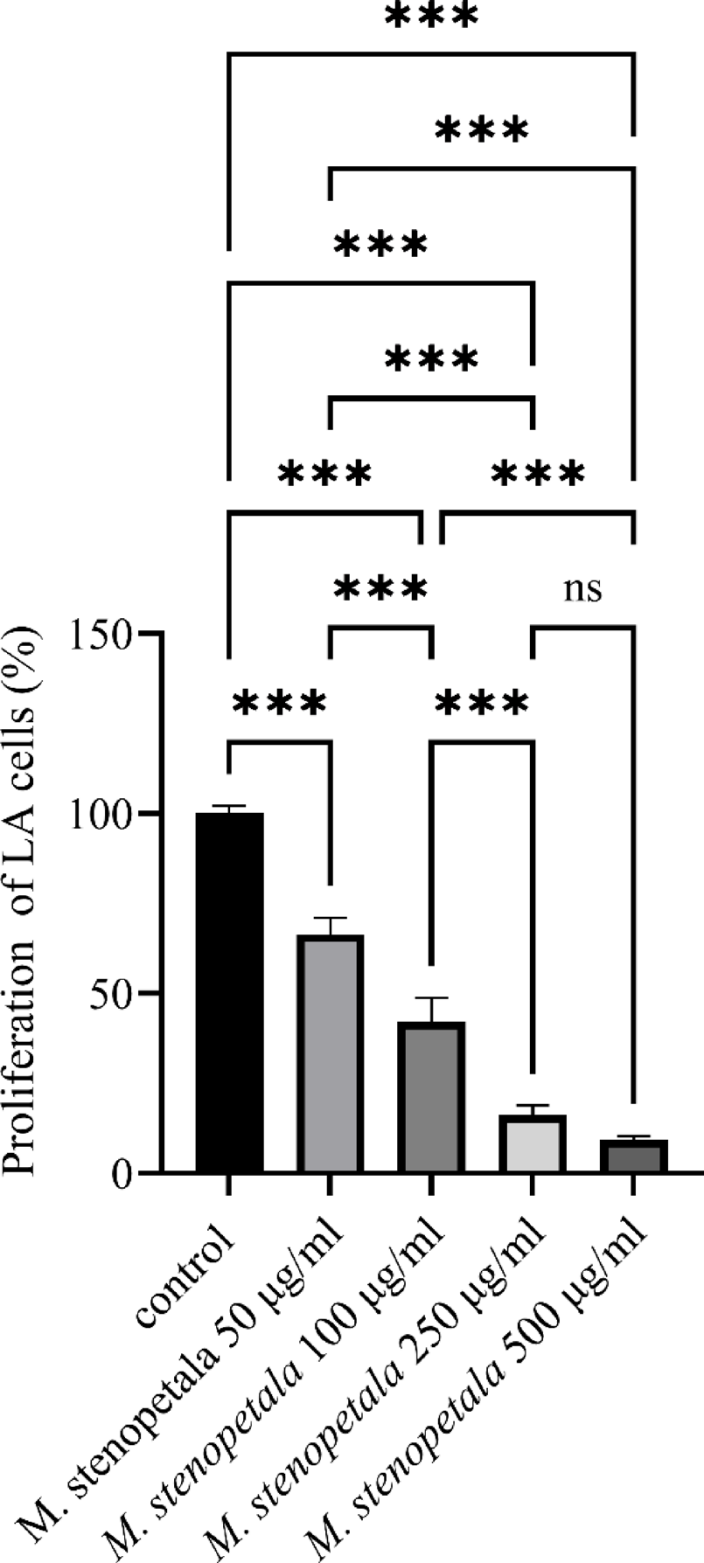
Proliferation of LA cells after the treatment with *M. stenopetala* leaf extract. Values are presented as mean ± SD (*n* = 3). Statistical significance was determined using one-way ANOVA followed by Tukey’s post-hoc test. Differences were considered significant at *P < 0.05, **P < 0.01, and ***P < 0.001; ns indicates non-significant (*P* > 0.05).

*M. stenopetala* extract was applied to LA cells at varying concentrations (50 µg/ml, 100 µg/ml, 250 µg/ml, and 500 µg/ml). Figure 5 illustrated that treatment with *M. stenopetala* extract decreased the proliferation of LA cells with increasing extract concentrations compared to the control. The results of the cell viability assay displayed that after 48 h of *M. stenopetala* leaf extract treatment (50–500 µg/ml), the proliferation of anaplastic thyroid carcinoma cells significantly decreased to 66.4%, 42.2%, 16.5%, and 9.3%, respectively, compared to the control. The decrease in percentage proliferation becomes significant with all doses of *M. stenopetala* extract (*P* < 0.001). The cell count verified the above-mentioned findings. In the control group of LA cells, the number of cells was 18,800 ± 920/100 µl/well; in the group treated with *M. oleifera* leaf extract with 50 µg/ml, 100 µg/ml, 250 µg/ml, and 500 µg/ml, the number of cells was 13,008 ± 970 (66%), 9,274 ± 760 (42%), 3,349 ± 460 (17%), and 1,611 ± 450 (9%), respectively (*P* < 0.01, ANOVA).

### Antiproliferative activity of the two species on the proliferation of LA cells

**Fig. 6:**
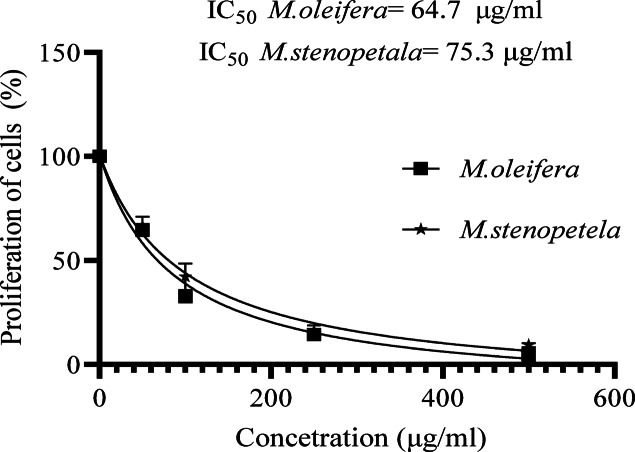
Comparative proliferation inhibition of *M. oleifera* and *M. stenopetala* on LA Cells. Values are presented as mean ± SD (*n* = 3). IC_50_ values were determined after 48 h of exposure. Although plant *M. oleifera* has a lower IC_50_ (64.7 µg/mL) than *M. stenopetala* (75.3 µg/mL), t-test revealed no significant difference (*P* > 0.05).

Increased concentrations of both *Moringa* species caused a dose-responsive decrease in cancer cell proliferation, as illustrated in Fig. 6. *M. oleifera* had a greater antiproliferative effect than *M. stenopetala*, but statistical t-test analysis revealed no significant differences (*P* > 0.05). *M. oleifera* leaf extract had a lower IC_50_ value (64.7 µg/ml) in LA cells after 48 h of exposure compared to *M. stenopetala* extract (75.3 µg/ml) (Fig. 6). Because of the lower IC_50_ value, *M. olefera (*IC_50_ = 64.7 µg/ml) has a greater cytotoxic effect than *M. stenopetala* (IC_50_ = 75.3 µg/ml) on cell viability.

## Discussion

Cancer is a disease where cells multiply uncontrollably and perform inappropriately^37^. LA accounts for approximately 40% of all lung malignancies and arises from the mucosal glands^6^. ATC is among the worst cancers in humans, with an average survival period of six to ten months^30^. Developing anticancer drugs from plants provides cost-effective, less toxic, and fewer side effects than conventional drugs^38^.

*M. oleifera’s* anticancer properties have been investigated in several cancer cell lines, including breast cancer^36,39^, colon cancer^40^, liver cancer^41^, and lung cancer^42,43^. Although numerous studies have examined *M. oleifera’s* impact on other cancer types, nothing is known about how it affects ATC, a rare but fatal form of thyroid cancer^44^. This study is among the first to assess how *M. oleifera* affects ATC, providing new information on how this plant can be used to treat thyroid cancer. Furthermore, while *M. oleifera’s* anticancer effect is well-documented, less is known about other *Moringa* species, including *M. stenopetala*, which is equally important and valuable^19^. Additionally, the effect of *Moringa* on primary cell cultures has not been previously reported. By assessing both species in primary ATC and LA cell cultures, this study addresses a significant gap and provides more information.

WST-1, like MTT (3-(4,5-dimethylthiazol-2-yl)-2,5-diphenyltetrazolium bromide), produces formazan dye through a reaction with the mitochondrial succinate-tetrazolium reductase. Furthermore, the WST-1 reagent yields a water-soluble formazan, and without the need for a separate solubilization step, the reaction product can be measured in 0.5 to 4 h. As a result, the WST-1 assay has gained popularity recently^45^. In comparison to MTT and XTT (2,3-bis[2 methoxy-4-nitro-5-sulfophenyl]-2 H-tetrazolium-5-carboxanilide), the WST-1 reagent has several advantages, including greater stability, rapidity, sensitivity, and water solubility^46^. Our study used the WST-1 assay in primary cell culture to demonstrate the antiproliferative properties of two *Moringa* species against ATC and LA cells. Cell proliferation was also verified by cell number counting.

Our findings showed that the leaf extracts of *M. oleifera* and *M. stenopetala* significantly inhibited the proliferation of ATC cells. The extracts’ bioactive ingredients and antioxidant qualities are probably responsible for this antiproliferative impact. Several studies have reported that *M. oleifera* leaves are particularly rich in antioxidants, such as carotenoids, α-tocopherol, vitamin C, flavonoids, and phenolics^47^. The antiproliferative effects of *M. oleifera* extracts on ATC cells may arise from various mechanisms. Previous research on *M. oleifera* demonstrates that its phytochemicals can induce apoptosis by activating caspase cascades, disrupting mitochondrial membrane potential, and elevating the production of reactive oxygen species (ROS), thereby inducing oxidative stress specifically in cancer cells^48,49^. In addition, these extracts have been shown to stop the cell cycle, especially at the G2/M phase, which stops the growth and division of cancer cells^50^. Similarly, *M. stenopetala* also has the highest concentration of secondary metabolites, including phenolics, flavonoids, tannins, and terpenoids^51,52^. *M. stenopetala* methanol extract LC-qTOF-MS profiling reveals 50 compounds, among which terpenes (loliolide and dihydroactinidiolide), niazirin, quinic acid derivatives, and flavonoids showed a potent cytotoxic effect^19^. These findings support the idea that the bioactive compounds and antioxidants in both *Moringa* species are crucial factors contributing to their observed antiproliferative effects on ATC cells. But as far as we are aware, no research has looked into the effects of the two *Moringa* species on ATC cells, emphasizing the originality of our findings in this context.

ATC cell proliferation was suppressed by two species, with IC_50_ values of 90.6 µg/ml (*M. stenopetala*) and 84.2 µg/ml (*M. oleifera*). Based on these IC_50_ values and the American NCI classification, these two species have moderate antiproliferative activity against LA cells^53^, indicating that these species could be viable candidates for future research into alternate therapeutics for ATC, a very aggressive and treatment-resistant malignancy.

Our findings also revealed that the leaf extracts of the two species significantly inhibited the proliferation of LA cells. This antiproliferative effect is most likely caused by the extracts’ bioactive components and antioxidant properties. Our findings are in line with earlier research on lung cancer cell lines. For example, the antiproliferative effect of *M. oleifera* on the lung cancer cell line has been well-reported^42,43^. However, no research has yet been conducted on the effects of *M. stenopetala* on lung cancer cell lines or the effects of the two species on primary cell culture, underscoring the uniqueness of our findings in this context.

LA cell proliferation was suppressed by two species, with IC_50_ values of 75.3 µg/ml (*M. stenopetala*) and 64.7 µg/ml (*M. oleifera*). According to these IC_50_ values and the American NCI classification^53^, both species have moderate antiproliferative activity against LA cells. The IC_50_ value of *M. oleifera* against LA cells is lower than that published by^54^, whose IC_50_ value of *M. oleifera* against A549 cell line was 166.7 µg/ml, and by^43^, whose IC_50_ of *M. oleifera* against A549 was 158.67 µg/ml, indicating that these two species may serve as a basis for future LA therapeutic applications.

The current study’s findings are promising; however, it is important to highlight that treating *Moringa* leaves with a limited range of solvents may influence the observed antiproliferative capabilities, as these effects can vary depending on the extraction method. Although our study used primary cell cultures, we recommend performing identical extractions in continuous cell cultures to compare our results. Furthermore, studies into how chemicals in *Moringa* extracts affect critical cancer-related processes such as apoptosis, cell cycle regulation, and oxidative stress response can yield useful results. In addition, validation in larger, more diverse cohorts and follow-up in vivo and clinical studies will be necessary to confirm the therapeutic potential and safety of the extracts.

## Conclusion

The current study demonstrates the antiproliferative effects of two *Moringa* species on ATC and LA cells in primary cell culture. Both *Moringa* species have antiproliferative activity on anaplastic thyroid carcinoma and lung adenocarcinoma cells. *M. oleifera* has a stronger effect than *M. stenopetala;* however, a statistical t-test analysis revealed no significant differences in the proliferation of cancer cells between the two species. These findings indicated that both extracts may serve as a basis for future cancer therapeutic applications. Despite the above uncertainties, the current results provide a strong foundation for future research into the use of *Moringa* species as natural anticancer agents. Therefore, promoting this kind of research should be encouraged to achieve the long-term objective of developing natural cancer treatments, which have the advantage of being safer, less expensive, and having fewer side effects.

## Data Availability

All data and materials are fully presented in the manuscript; further requests can be directed to the corresponding authors.
